# Perceived Importance of Physical Activity and Walkable Neighborhoods Among US Adults, 2017

**DOI:** 10.5888/pcd17.200262

**Published:** 2020-12-31

**Authors:** Susan A. Carlson, Emily N. Ussery, Kathleen B. Watson, Kelly A. Cornett, Janet E. Fulton

**Affiliations:** 1 Physical Activity and Health Branch, Division of Nutrition, Physical Activity, and Obesity, National Center for Chronic Disease Prevention and Health Promotion, Centers for Disease Control and Prevention, Atlanta, Georgia

## Abstract

The importance of physical activity and community-level promotion strategies are well established, but little is known about adult perception of the importance of physical activity. In a nationwide sample of US adults, we examined self-reported importance of regular physical activity and the importance of living in walkable neighborhoods. About 55% of adults strongly agreed that regular physical activity is important, 40% strongly agreed that living in a walkable neighborhood is important, and 31% strongly agreed that both are important. Separately for each measure, estimates were lower among adults with lower education levels and who did not meet the aerobic physical activity guideline. Opportunities exist to improve the perception of the importance of physical activity and the importance of walkable neighborhoods.

**SUMMARY**

**What is already known on this topic?**

Regular physical activity can produce long-term health benefits. A key supportive strategy for physical activity is walkable neighborhoods.

**What is added by this report?**

In a nationwide sample of US adults, we examined self-reported importance of regular physical activity and living in a walkable neighborhood. The prevalence of strong agreement with importance was 55% for regular physical activity, 40% for living in a walkable neighborhood, and 31% for both. Similar results were found across education levels and physical activity behavior.

**What are the implications for public health practice?**

Opportunities exist to improve the perceived importance of regular physical activity and walkable neighborhoods, especially among those who are least active.

## Objective

Regular physical activity can produce long-term health benefits (1). Evidence-based strategies exist for promoting physical activity, including the creation of walkable communities, where people can safely and easily walk for transportation, relaxation, or exercise (2). Several national initiatives promote the benefits of physical activity and walkable communities (2–5). A better understanding of how the perception of importance differs by individual characteristics can help guide physical activity initiatives. We examined the self-reported importance of regular physical activity and of living in walkable neighborhoods by demographic characteristics and by physical activity behavior in a nationwide sample of adults.

## Methods

Porter Novelli’s 2017 Styles (https://styles.porternovelli.com) database gathers data on US consumer insights, including information about health attitudes and behaviors. The panel has about 55,000 panelists. The initial SpringStyles survey was sent during March–April 2017 to 10,916 panelists; of these, 6,622 completed the survey (response rate, 60.7%). The summer wave, SummerStyles, was sent during June–July to 5,586 adults who completed the SpringStyles survey; of these, 4,107 completed the survey (response rate: 73.5%). Respondents received a small incentive of about $10.

Respondents were asked how strongly they disagree or agree (ie, strongly disagree, somewhat disagree, neither agree or disagree, somewhat agree, or strongly agree) with the following statements: 1) I think it is important to be physically active on a regular basis, and 2) I think it is important to live in a neighborhood that is walkable. We defined a walkable neighborhood as one that it is safe and easy for people to walk to get somewhere or one that is safe and easy to walk for fun, relaxation, or exercise. Given the small percentage of respondents reporting disagreement, we tabulated estimates for 3 levels of agreement: 1) disagree and neither agree nor disagree, 2) somewhat agree, and 3) strongly agree.

Respondent demographic characteristics included sex, age, education, race/ethnicity, region (6), and metropolitan statistical area (MSA) status (7). Respondents were classified as meeting the aerobic physical activity guideline (≥150 min/wk of moderate-intensity–equivalent physical activity) or not meeting the aerobic physical activity guideline (1).

Of the 4,107 SummerStyles respondents, 3,991 had complete data for all variables of interest. Using these cross-sectional data, we examined the importance of regular physical activity and the importance of living in a walkable neighborhood by demographic characteristics. We examined separate and combined importance by physical activity levels. Analyses were conducted using SUDAAN, Release 11 (RTI International) to account for survey weights. Within subgroups, we separately tested pairwise differences in the prevalence of 3 agreement levels using adjusted Wald χ^2 ^tests with a Bonferroni correction. Trends in prevalence of strong agreement by age and education were tested with orthogonal polynomial contrasts. Results with *P* value <.05 were considered significant.

## Results

Overall, an estimated 54.7% of adults (unweighted n = 2,216 respondents) strongly agreed that it is important to be regularly physically active (Table). Women compared with men, adults aged 50 or older compared with adults aged 18 to 34, and those who met the aerobic guideline compared with those who did not were more likely to strongly agree that it is important to be regularly physically active. Reports of strong agreement with the importance of regular physical activity increased linearly as education increased (*P* <.001).

**Table:** Self-Reported Importance of Regular Physical Activity and Living in a Walkable Neighborhood by Select Characteristics, Adults (N = 3,991), SummerStyles 2017

Characteristic	No.^b^	Importance of regular physical activity^a^			Importance of living in a walkable neighborhood^a^		
		Disagree/Neither Agree nor Disagree, % (95% CI)	Agree% (95% CI)		Disagree/Neither Agree nor Disagree % (95% CI)	Agree % (95% CI)	
			Somewhat	Strongly		Somewhat	Strongly
**Overall**	3,991	15.0 (13.7–16.4)	30.3 (28.7–31.9)	54.7 (52.9–56.4)	27.3 (25.7–28.9)	33.0 (31.4–34.7)	39.7 (38.0–41.4)
**Sex**							
Male	1,937	16.9 (15.0–19.1)	32.1 (29.8–34.5)	51.0 (48.4–53.5)	28.3 (26.0–30.7)	33.9 (31.5–36.3)	37.8 (35.4–40.3)
Female	2,054	13.2 (11.6–15.0)	28.7 (26.5–30.9)	58.1 (55.7–60.5)	26.3 (24.3–28.5)	32.3 (30.1–34.5)	41.4 (39.1–43.8)
**Age, y**							
18–34	844	20.7 (17.8–24.0)	30.2 (26.9–33.6)	49.1 (45.4–52.8)	30.5 (27.2–34.1)	32.9 (29.5–36.4)	36.6 (33.1–40.3)
35–49	1,094	17.2 (14.7–20.1)	27.1 (24.3–30.1)	55.7 (52.4–59.0)	29.0 (26.1–32.2)	31.0 (28.1–34.1)	39.9 (36.8–43.2)
50–64	1,306	11.2 (9.4–13.2)	30.3 (27.7–33.1)	58.5 (55.6–61.4)	26.3 (23.8–28.9)	34.2 (31.5–37.1)	39.5 (36.6–42.4)
≥65	747	8.9 (6.8–11.5)	34.6 (31.0–38.3)	56.6 (52.7–60.4)	21.7 (18.6–25.2)	34.1 (30.5–37.8)	44.3 (40.4–48.2)
**Race/ethnicity**							
Non-Hispanic White	2,923	13.3 (11.9–14.7)	31.3 (29.5–33.1)	55.5 (53.5–57.4)	27.1 (25.3–28.9)	34.6 (32.8–36.5)	38.3 (36.4–40.3)
Non-Hispanic Black	360	22.3 (17.7–27.6)	26.3 (21.6–31.6)	51.5 (45.8–57.1)	34.0 (28.8–39.6)	24.9 (20.3–30.1)	41.1 (35.7–46.7)
Other^c^	708	16.2 (13.3–19.6)	29.6 (26.0–33.5)	54.2 (50.0–58.3)	24.5 (21.1–28.3)	32.7 (29.0–36.7)	42.7 (38.7–46.8)
**Education**							
Some high school	1,487	21.9 (19.4–24.6)	32.5 (29.9–35.2)	45.6 (42.7–48.5)	33.5 (30.8–36.3)	31.6 (29.0–34.3)	34.9 (32.2–37.8)
Some college	1,202	11.9 (10.1–14.1)	32.4 (29.5–35.4)	55.7 (52.5–58.8)	25.8 (23.1–28.6)	34.5 (31.6–37.6)	39.7 (36.6–42.8)
College graduate	1,302	9.0 (7.4–10.9)	25.5 (23.0–28.2)	65.5 (62.6–68.3)	20.7 (18.3–23.2)	33.5 (30.8–36.3)	45.8 (42.9–48.8)
**Census region**							
Northeast	770	16.1 (13.3–19.3)	27.0 (23.7–30.5)	56.9 (53.0–60.8)	26.2 (23.0–29.8)	32.7 (29.2–36.5)	41.0 (37.2–45.0)
Midwest	873	14.7 (12.1–17.7)	32.5 (29.1–36.0)	52.8 (49.1–56.6)	28.0 (24.7–31.6)	33.4 (30.0–37.0)	38.6 (35.1–42.3)
South	1,454	14.8 (12.7–17.1)	32.8 (30.2–35.6)	52.4 (49.5–55.3)	28.8 (26.2–31.5)	33.4 (30.8–36.1)	37.8 (35.1–40.7)
West	894	14.8 (12.3–17.8)	26.9 (23.8–30.3)	58.3 (54.5–61.9)	25.1 (21.9–28.5)	32.4 (29.0–35.9)	42.6 (39.0–46.2)
**MSA status^d^**							
Nonmetro	573	14.1 (11.0–18.0)	33.0 (29.0–37.4)	52.9 (48.3–57.4)	33.2 (28.9–37.7)	32.9 (28.9–37.3)	33.9 (29.6–38.4)
Metro	3,418	15.2 (13.8–16.7)	29.9 (28.2–31.6)	55.0 (53.1–56.9)	26.3 (24.7–28.0)	33.0 (31.3–34.8)	40.6 (38.8–42.5)
**Aerobic physical activity guideline^e^**							
Does not meet	1,629	19.1 (16.9–21.4)	41.2 (38.6–44.0)	39.7 (37.0–42.4)	32.3 (29.7–34.9)	33.3 (30.8–35.9)	34.4 (31.8–37.0)
Meets	2,362	12.1 (10.6–13.8)	22.6 (20.8–24.5)	65.3 (63.1–67.4)	23.8 (21.9–25.8)	32.8 (30.7–35.0)	43.4 (41.2–45.7)

Abbreviation: MSA, metropolitan statistical area.

a Some values for responses to statements are Bonferroni adjusted, *P* <.05.

b Estimates were weighted using survey weights provided with the data set. Weights were created to match US Current Population Survey proportions for sex, age, household income, race/ethnicity, household size, education, census region, and MSA status before joining the panel. Of 4,107 respondents (unweighted), 116 were excluded for missing data on importance of regular physical activity or living in a walkable community of physical activity level. Unweighted sample sizes by level of agreement in the importance of regular physical activity: 106 disagree strongly; 66 disagree somewhat; 368 neither agree nor disagree; 1,235 somewhat agree; and 2,216 strongly agree. Sample sizes by level of agreement in the importance of a walkable neighborhood: 98 disagree strongly; 162 disagree somewhat; 800 neither agree nor disagree; 1,371 somewhat agree; and 1,560 strongly agree.

c Other race/ethnicity includes Hispanic, American Indian or Alaska Native, Asian, and Native Hawaiian and Other Pacific Islander.

d A respondent was defined as living in a metro area if that area is in a core-based statistical area that was associated with at least 1 urbanized area having a population of at least 50,000.

e Physical activity was assessed by asking how often and for how long the respondent participated in vigorous- and moderate-intensity activities for at least 10 minutes at a time during leisure time in a usual week. Total volume of moderate-intensity–equivalent physical activity (min/wk) was calculated as weekly frequency × duration with each minute of vigorous-intensity physical activity multiplied by 2. Total physical activity was classified in 2 categories: meets the aerobic guideline (≥150 min/wk of moderate-intensity–equivalent activity) and does not meet the aerobic guideline.

Overall, 39.7% of adults (unweighted n = 1,560) strongly agreed it is important to live in a walkable neighborhood (Table). Women compared with men, adults who lived in a metropolitan area compared with those living in a nonmetropolitan area, and those who met the aerobic guideline compared with those who did not were more likely to strongly agree that it is important to live in a walkable neighborhood. Prevalence of strong agreement increased linearly as age group (*P* = .007) and education (*P* <.001) increased. 

Overall, 31.2% of adults (unweighted n = 1,227) strongly agreed that it is important to be regularly physically active and live in a walkable neighborhood (Figure). Prevalence of strong agreement that it is important to be regularly physically active and live in a walkable neighborhood was higher among adults meeting the aerobic guideline (37.5%; 95% CI, 35.3%–39.7%) than among those who do not meet the guideline (22.2%; 95% CI, 20.0%–24.6%).

**Figure:**
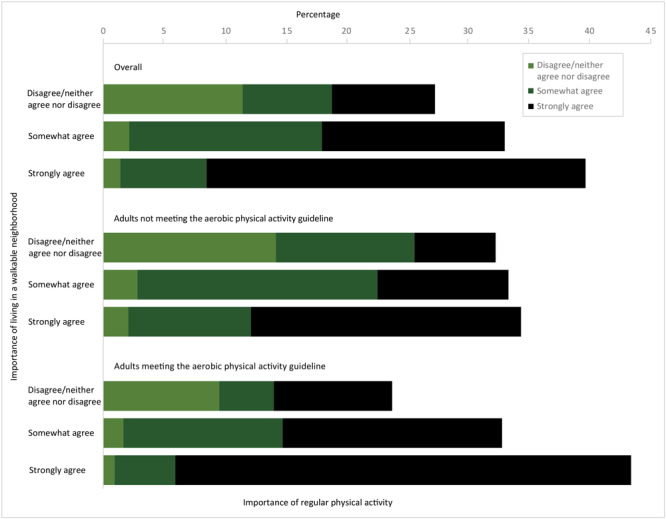
Self-reported importance of regular physical activity and of living in a walkable neighborhood for adults overall, for adults meeting the aerobic physical activity guideline, and for adults not meeting the guideline, SummerStyles 2017.

**Table:** 

Statement Response	Disagree/Neither Agree nor Disagree, % (95% CI)	Agree	
		Somewhat Agree, % (95% CI)	Strongly Agree, % (95% CI)
**Overall** “I think it is important to live in a walkable neighborhood”			
With importance of regular physical activity —Disagree/Neither Agree nor Disagree	11.5 (10.3–12.7)	2.1 (1.7–2.7)	1.4 (1.0–1.9)
With importance of regular physical activity —Somewhat Agree	7.4 (6.5–8.3)	15.8 (14.6–17.2)	7.1 (6.3– 8.0)
With importance of regular physical activity —Strongly Agree	8.5 (7.6–9.5)	15.0 (13.9–16.3)	31.2 (29.6–32.8)
**Do not meet aerobic guideline** “I think it is important to live in a walkable neighborhood”			
With importance of regular physical activity —Disagree/Neither Agree nor Disagree	14.2 (12.3–16.4)	2.8 (2.1–3.8)	2.1 (1.4–3.1)
With importance of regular physical activity —Somewhat Agree	11.4 (9.8–13.2)	19.8 (17.7–2.0)	10.1 (8.6–11.8)
With importance of regular physical activity —Strongly Agree	6.7 (5.4–8.1)	10.8 (9.2, 12.5)	22.2 (20.0–24.6)
**Meet aerobic guideline** “I think it is important to live in a walkable neighborhood”			
With importance of regular physical activity —Disagree/Neither Agree nor Disagree	9.5 (8.2–11.1)	1.7 (1.1–2.5)	0.9 (0.6–1.5)
With importance of regular physical activity —Somewhat Agree	4.5 (3.7–5.5)	13.1 (11.6–14.7)	5.0 (4.1–6.1)
With importance of regular physical activity —Strongly Agree	9.7 (8.5–11.1)	18.1 (16.4–19.8)	37.5 (35.3–39.7)

## Discussion

An estimated 55% of adults strongly agreed that it is important to engage in regular physical activity, 40% strongly agreed that a walkable neighborhood is important, and 31% strongly agreed that both are important. Similar to our findings, an earlier study estimated 52% of adults identified physical inactivity as an important risk factor, and higher physical activity levels were generally associated with greater perceived importance (8). Some subgroups identified as less physically active in an earlier study, including those with lower education status (9), were also less likely to strongly agree on the importance of our measures. Tailoring promotion strategies for subgroups may be important to consider (10). For example, organizations can provide access to programs that help participants overcome barriers to participation, and campaign activities can be designed to resonate with specific audiences (2).

Multiple national initiatives promote the benefits of physical activity and walkable communities. Move Your Way, the promotional campaign for the Physical Activity Guidelines for Americans, 2nd edition, provides materials highlighting the importance of physical activity (1,4). Step It Up, The Surgeon General’s Call to Action to Promote Walking and Walkable Communities, calls on Americans and partners working across sectors to support walking and walkability for people of all ages and abilities (2). For example, people can participate in planning processes to support walkability in their communities, and the transportation, land use, and community design sectors can help to ensure an equitable distribution of the benefits of walkability (2). Active People, Healthy Nation, an initiative led by the Centers for Disease Control and Prevention to help 27 million Americans become more physically active by 2027 (3,5), encourages people to explore the health benefits of physical activity and learn how to be a part of creating a more active America (5). Several evidence-based strategies to improve walkability in communities are highlighted in the initiative, including activity-friendly routes to everyday destinations (5,11).

Our study has at least 2 limitations. First, sample selection bias might be associated with use of data from a panel of volunteers, which might limit generalizability of our results. Weighting our data to US Census Bureau proportions, however, might help address this concern. Second, physical activity was self-reported, and reporting bias may have resulted in overestimates of physical activity (12). 

The health benefits of physical activity are well established. Our findings suggest that opportunities exist to strengthen adult perceptions about the importance of regular physical activity and walkable neighborhoods, especially among adults who do not meet the aerobic physical activity guideline.
